# Campho-Phenique

**Published:** 1891-08

**Authors:** 


### Campho-Phenique.

As day by day this valuable preparation grows in the knowledge of physicians and surgeons, so does it grow in their esteem. It has now testimonials of the highest character from those who have tried it, and is unquestionably one of the best surgical dressings ever offered to the profession. Dr. M. D. Hoge, of Richmond, Va., says that it effectually dissolves and checks the extension of the diphtheritic membrane, and is easily applied without dilution.—*Toledo Medical and Surgical Reporter.*

